# Food Markets with Live Birds as Source of Avian Influenza

**DOI:** 10.3201/eid1211.060675

**Published:** 2006-11

**Authors:** Ming Wang, Biao Di, Duan-Hua Zhou, Bo-Jian Zheng, Huaiqi Jing, Yong-Ping Lin, Yu-Fei Liu, Xin-Wei Wu, Peng-Zhe Qin, Yu-Lin Wang, Li-Yun Jian, Xiang-Zhong Li, Jian-Xiong Xu, En-Jie Lu, Tie-Gang Li, Jianguo Xu

**Affiliations:** * Guangzhou Center for Disease Control and Prevention, Guangzhou, People's Republic of China;; † The University of Hong Kong, Hong Kong Special Administrative Region, People's Republic of China;; ‡ National Institute for Communicable Disease Control and Prevention, Beijing, People's Republic of China;; § State Key Laboratory for Infectious Disease Prevention and Control, Beijing, People's Republic of China

**Keywords:** Highly pathogenic avian virus, food markets, live birds, dispatch

Highly pathogenic avian influenza virus (HPAI) H5N1 infected 202 persons worldwide and killed 113 as of April 30, 2006 (*1*). Most patients were exposed to ill or dead birds or were involved in the slaughter or preparation of birds for human food (*2*). However, of 19 patients with confirmed cases in the People's Republic of China, 5 had no history of direct contact with ill or diseased birds and resided in urban or periurban areas that did not have farmed birds. We studied an infected patient from Guangzhou who did not report contact with birds.

## The Study

The patient was from Guangzhou, the capital of the southern province of Guangdong. A fever (39°C) developed on February 22, 2006. He was hospitalized on February 26 and died on March 2. Diagnosis of influenza virus infection was made on March 3. Throat swab specimens obtained on March 1 and 2 tested positive for HPAI H5N1 virus by reverse transcription (RT)–PCR. Virus was isolated and named A/Guangzhou/1/2006 (H5N1).

Epidemiologic studies showed that the patient did not slaughter, process, or cook birds. However, while looking for work before his illness, he visited 9 food markets that had live birds. All 9 markets were located in the central part of the city (Table). He visited food market F twice a day from January 23 to 27 and food market G on February 17 for 30 minutes. Before his illness, he and his girlfriend (whom he lived with) shopped at markets B and F on February 20–22. He also visited food market I from February 10 to February 20. The dates he visited the other food markets could not be determined. Onset of fever occurred on February 22.

**Table:** Serum and swab sample results from live birds and animal cages sampled at markets in Guangzhou, People's Republic of China*

Source	Food market																			
	A		B		C		D		E		**F**		G		H		**I**			
	T	S	T	S	T	S	T	S	T	S	T	S	T	S	T	S	T	S	T	S
Serum																				
Poultry purveyors	22	22	22	21	6	5	2	2	14	12	**14**	13	14	14	12	11	15	10	121	110
Swabs																				
Animal cages	20	10	27	0	8	8	4	0	16	3	28	8	27	18	24	8	27	**24**	181	79
Anal swabs																				
Chicken	160	10	190	6	64	6	36	5	95	6	268	7	195	3	160	1	205	13	1,373	57
Duck	10	0	5	1	4	1	0	0	0	0	3	1	3	3	2	1	10	5	37	12
Goose	5	0	0	0	0	0	0	0	3	1	3	1	0	0	3	1	6	4	20	7
Pigeon	15	0	20	3	10	2	0	0	0	0	0	0	15	2	10	1	70	5	140	13
Partridge	30	0	45	0	30	1	0	0	0	0	0	0	0	0	10	1	20	2	135	4
Quail	80	0	110	0	60	0	0	0	0	0	0	0	0	0	0	0	15	1	265	1

*T, total no.; S, no. sampled. Markets and samples from which specimens tested positive for virus genes or neutralizing antibody against highly pathogenic avian influenza virus H5N1 are in **boldface**.

The food markets were typically large, clean, and well managed and had vendors selling vegetables, fruits, raw and cooked meats, food flavorings, beverages, and other goods. They are typical of larger food markets in cities in the People's Republic of China. The only difference between markets in Guanzhou in southern China and those in cities in northern China is that more (*2**–**9*) booths are used to sell live birds in Guanzhou. Wire cages are stacked next to each other with ≈5–10 birds in each cage (chickens, geese, ducks, and pigeons). Each species of bird is placed in separate cages; chickens are the most common species. All cages are located in a closed room separated by a glass window from customers, who choose the bird they prefer. When a live bird is selected, it is slaughtered in view of the customer. Sanitation inspections are routinely performed by municipal authorities. No diseased or dead birds were observed during this investigation.

**Figure 1:**
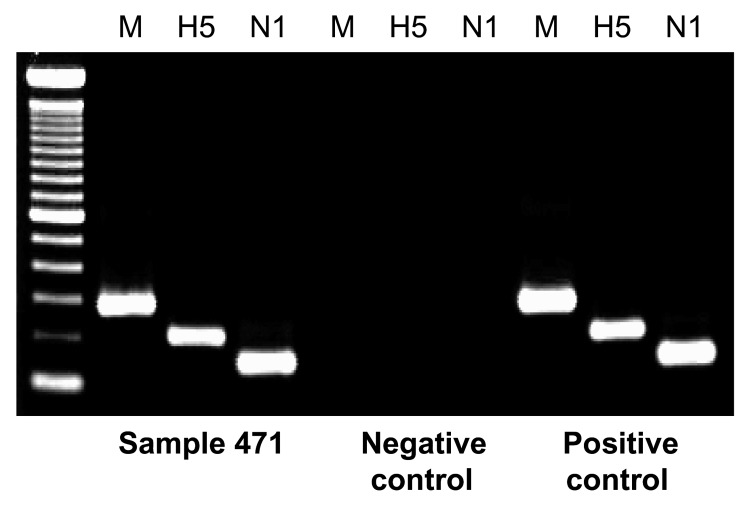
Detection of avian influenza virus H5N1 from an animal cage for geese by reverse transcription–PCR. Viral RNA was extracted from the sample and amplified by using 3 pairs of primers specific for membrane (M), hemagglutinin (H5), and neuraminidase (N1) virus genes. Sample buffer was used as a negative control, and viral RNA from a human H5N1 virus strain (A/Hong Kong/486/97) was included as a positive control. First lane, molecular mass ladder.

**Figure 2:**
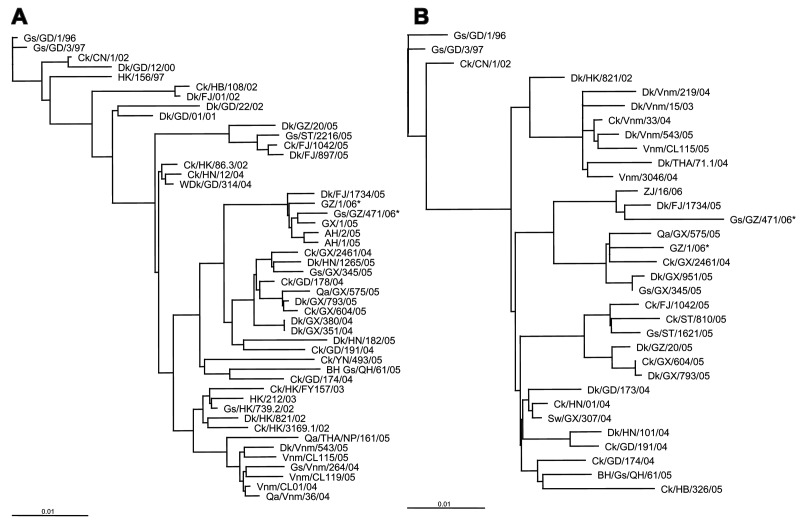
Phylogenetic relationships of representative H5N1 influenza virus strains and patient and animal cage isolates (indicated by asterisks) used in this study. A) Hemagglutinin gene (nt positions 29–1650). B) Neuramidase gene (nt positions 28–1323). Gs, goose; GD, Guangdong; Ck, chicken; CN, People's Republic of China; Dk, duck; HK, Hong Kong; HB, Hebei; FJ, Fujian; GZ, Guangzhou; ST, Shantou; HN, Hunan; WDK, wild duck; GX, Guangxi; AH, Anhui; Qa, quail; YN, Yunnan; BH Gs, brown-headed goose; QH, Qinghai; THA, Thailand, NP, Nakhon Pathom; Vnm, Vietnam; ZJ, Zhejiang; Sw, swine. Scale bars show percentage relatedness.

Animal cages were swabbed and anal swabs of live birds were obtained at the food markets (Table) on March 3 and 4 and tested for HPAI by using RT-PCR (*3*) for the hemagglutinin (H5), neuraminidase (N1), and membrane (M) genes. Positive PCR results were confirmed by sequencing. None of 94 anal swabs from live birds tested positive for HPAI H5N1. However, 1 of 79 animal cage swabs tested positive for HPAI H5N1 (Figure 1). The positive swab was from a goose cage at market I (Table), the market that the patient visited from February 10 to February 20. The nucleotide sequences of H and M genes from specimens from this patient were compared with those from the animal cage swab and submitted to GenBank (accession nos. DQ842487–90). Forty-eight variations were found in the NA gene and 15 were found in the HA gene, which resulted in 17 HA amino acid and 3 NA amino acid changes, respectively. Phylogenetic analysis with the neighbor-joining method using the ClustalX program (*4*) suggested that the 2 strains are related to each other and to duck isolates (Figure 2).

Serum samples were obtained from 110 of 121 poultry purveyors working at the live bird food markets and screened for antibody to H5N1 to determine if subclinical infections occurred. One of 110 serum samples was positive (titer 320) by hemagglutination-inhibition assay with turkey erythrocytes (Lampire Biologic Laboratories, Pipersville, PA, USA) and H5N1 virus strains A/Hong Kong/486/97 and A/Vietnam/1194/04/H5N1 (*5*). Neutralizing antibody titers against the 2 strains of virus were 1,280 and 640, respectively. The positive serum sample was from a 44-year-old man who slaughtered birds for 5 years. He slaughtered ≈100 chickens/day and did not report any recent respiratory diseases. He denied any contact with ill birds.

## Conclusions

Our investigation suggests that the patient may have been infected by an unknown mechanism at a food market that had live birds. We detected H5N1 virus genes in a swab from a goose cage and neutralizing antibody against H5N1 in a poultry worker in 1 of the food markets the patient visited.

This case from Guangzhou was not an isolated event. Five patients with H5N1 infection with no history of exposure to diseased or dead birds before the onset of avian influenza have been reported in the People's Republic of China; 4 of these 5 patients visited markets that had live birds. The first patient was a 41-year-old woman from Fuzhou, the capital of Fujian Province, whose diagnosis was made in December 2005 (*6*). She visited a market that had live birds 2 weeks before her illness. Another patient lived in a periurban area of Chengdu, the capital of Sichuan Province; her diagnosis was made in January 2006. She was self-employed in a shop selling dry goods at a market that had live birds in Jinhua Town in Chengdu (*7*). Two other patients in urban areas were reported, 1 in Shanghai and 1 in Shenzhen. Influenza was diagnosed in the patient in Shanghai in March 2006, but this patient had no history of visiting a food market that had live birds or contact with diseased birds (*8*). Influenza was diagnosed in the patient in Shenzhen in April 2006; this patient reported visiting a food market that had live poultry before becoming infected with influenza virus.

Our findings suggest that food markets or farmers' markets that have live poultry may be a source for avian influenza infection in which healthy live birds may carry the virus. This was previously shown in Hanoi, Vietnam, in 2001, where H5N1 virus was detected in domestic birds in a live bird market (*9*). Serologic investigation also demonstrated low seroprevalence of antibody against HPAI H5N1 in poultry workers from this market. However, no outbreaks of HPAI among birds were reported until early 2004 (*10*). H5N1 virus may be sustained in poultry largely through the movement of poultry and poultry products, especially through domestic ducks (*11**,**12*). The introduction of H5N1 virus from healthy poultry (such as ducks) may be occurring where no outbreaks in healthy flocks have been observed. Therefore, the virus is likely reintroduced at low levels and can infect persons visiting live poultry markets.

The cultural preference of eating freshly slaughtered birds is not unique to the People's Republic of China; it is also common in other Asian countries. Our results suggest that the practice of selling live birds directly to consumers in food markets should be discouraged in areas currently experiencing influenza outbreaks among birds, especially in large modern cities where there may be a threat to the casual market visitor (*2**,**13**,**14*).

## References

[1] Epidemiology of WHO-confirmed human cases of avian influenza A(H5N1) infection. Wkly Epidemiol Rec. 2006;81:249–57. 16812929

[2] Thorson A, Petzold M, Nguyen TK, Ekdahl K. Is exposure to sick or dead poultry associated with flulike illness?: a population-based study from a rural area in Vietnam with outbreaks of highly pathogenic avian influenza. Arch Intern Med. 2006;166:119–23. 10.1001/archinte.166.1.119 16401820

[3] Payungporn S, Phakdeewirot P, Chutinimitkul S, Theamboonlers A, Keawcharoen J, Oraveerakul K, Single-step multiplex reverse transcription-polymerase chain reaction (RT-PCR) for influenza A virus subtype H5N1 detection. Viral Immunol. 2004;17:588–93. 10.1089/vim.2004.17.588 15671756

[4] Thompson JD, Gibson TL, Plewniak F, Jeanmougin F, Higgins DG. The CLUSTAL_X windows interface: flexible strategies for multiple sequence alignment aided by quality analysis tools. Nucleic Acids Res. 1997;25:4876–82. 10.1093/nar/25.24.4876 9396791 PMC147148

[5] Yu H, Shu Y, Hu S, Zhang H, Gao Z, Chen H, The first confirmed human case of avian influenza A (H5N1) in Mainland China. Lancet. 2006;367:84. 10.1016/S0140-6736(05)67894-4 16399159

[6] World Health Organization. Avian influenza situation in China, update 51. 2005 [cited 2006 Aug 21]. Available from http://www.who.int/csr/don/2005_12_30/en/

[7] World Health Organization. Avian influenza situation in China, update 2. 2006 [cited 2006 Aug 20]. Available from http://www.who.int/csr/don/2006_01_25a/en/index.html

[8] World Health Organization. Avian influenza situation in China, update 8. 2006 [cited 2006 Aug 20]. Available from http://www.who.int/csr/don/2006_03_24c/en/index.html

[9] Nguyen DC, Uyeki TM, Jadhao S, Maines T, Shaw M, Matsuoka Y, Isolation and characterization of avian influenza viruses, including highly pathogenic H5N1, from poultry in live bird markets in Hanoi, Vietnam, in 2001. J Virol. 2005;79:4201–12. 10.1128/JVI.79.7.4201-4212.2005 15767421 PMC1061558

[10] Tran TH, Nguyen TL, Nguyen TD, Luong TS, Pham PM, Nguyen VC, Avian influenza A (H5N1) in 10 patients in Vietnam. N Engl J Med. 2004;350:1179–88. 10.1056/NEJMoa040419 14985470

[11] Li KS, Guan Y, Wang J, Smith GJ, Xu KM, Duan L, Genesis of a highly pathogenic and potentially pandemic H5N1 influenza virus in eastern Asia. Nature. 2004;430:209–13. 10.1038/nature02746 15241415

[12] Chen H, Smith GJ, Li KS, Wang J, Fan XH, Rayner JM, Establishment of multiple sublineages of H5N1 influenza virus in Asia: implications for pandemic control. Proc Natl Acad Sci U S A. 2006;103:2845–50. 10.1073/pnas.0511120103 16473931 PMC1413830

[13] Martin V, Sims L, Lubroth J, Pfeiffer D, Slingenbergh J, Domenech J. Epidemiology and ecology of highly pathogenic avian influenza with particular emphasis on South East Asia. Dev Biol (Basel). 2006;124:23–36. 16447491

[14] Swayne DE. Occupational and consumer risks from avian influenza viruses. Dev Biol (Basel). 2006;124:85–90. 16447498

